# Anterior capsular morphologic changes in osteonecrosis of the femoral head linked to impaired quality of life and range of motion

**DOI:** 10.1097/MD.0000000000045779

**Published:** 2025-11-28

**Authors:** Anli Shi, Bobo Wang, Zhiqiang Wang, Zhaohui Ge

**Affiliations:** a Gansu Provincial Hospital, Lanzhou, China; b The First Clinical Medical College of Ningxia Medical University, Yinchuan, China; c Henan Provincial Hospital, Zhengzhou, China.

**Keywords:** anterior capsule, osteonecrosis, pathoanatomy

## Abstract

Pathoanatomical alterations in the anterior capsule of patients with osteonecrosis of the femoral head (ONFH) have not yet been identified. This study aimed to evaluate differences in the thickness and morphology of the anterior capsule and to explore further associations between them and quality of life. A total of 83 hips from 46 patients with ONFH were consecutively recruited, while 22 hips from 11 age- and sex-matched volunteers were collected as controls from January 2024 to September 2024. Participants were separated into 4 stages according to the Association Research Circulation Osseous classification. The thickness and morphology of the anterior capsule were assessed on the axial plane and coronal and axial planes by magnetic resonance imaging. Correlated analysis was subsequently performed between pathoanatomical alterations and quality of life scores, including the Short-Form-12-item Health Survey Version 2 (SF12) and the Oxford Hip Score (OHS), and range of motion (ROM). The anterior capsule thickness was significantly greater in stages III and IV patients than in stages I and II patients and controls (*P* < .001). Morphological alterations, including edema and delamination, were significant different at various stages (*P* < .001). The correlation between thickness and SF12, OHS, and ROM revealed moderate negative and morphological alterations showed similar outcomes. Patients with advanced-stage ONFH have thicker and more predominantly morphologically altered anterior capsules than patients in the initial stages. The thickness and morphological alterations both had a moderate negative correlation with SF12, OHS, and ROM.

## 1. Introduction

Osteonecrosis of the femoral head (ONFH) is characterized by an interruption of blood supply to the femoral head. This interruption progressively leads to subchondral fracture, femoral head collapse, and secondary osteoarthritis, resulting in severe pain and dysfunction, particularly in the terminal stage.^[1,2]^ Pathological changes in the hip joints of patients with ONFH directly affect hip function. Previous research has revealed definite histopathological disorders in the head.^[3,4]^ However, relatively little research has explored the pathoanatomical alterations of adjacent capsules in ONFH, while studies on other diseases are abundant.^[5–7]^

Componential abnormalities, such as a low proportion of type I collagen in the ligamentous capsule, have been suggested to be positively correlated with developmental dysplasia of the hip.^[8]^ Furthermore, early experiments of the hip joint showed that the anterior capsule was larger in cross-sectional area and more robust than the posterior capsule while also playing a vital role in stabilizing and flexing the hip.^[9,10]^ In addition to in vitro studies, additional imaging investigations have been performed evolvedly to assess the dimensions of the capsule in pathologic hip joints by magnetic resonance imaging (MRI) and have confirmed that the dimensions can be modified with progressive arthritis, which gradually leads to a larger cross-sectional area and tighter capsule.^[11,12]^ Unfortunately, the componential and dimensional alterations of the anterior capsule in patients with ONFH remain unclear.

Quality of life (QOL), comprising physical and mental function and range of motion (ROM) of the hip, has been proposed to be significantly different in patients with various magnitudes of femoral head collapse.^[13,14]^ Nevertheless, the association between QOL and pathoanatomical changes in the anterior capsule has not been studied in detail. The purpose of this study was to utilize MRI to observe preoperative alterations in the dimensions and components of the anterior joint capsule and to explore the relationship between its componential and dimensional changes and QOL in patients at various stages of ONFH.

## 2. Materials and methods

### 2.1. Patients

Initially, 96 hips were collected, of which 83 hips from 46 patients met the inclusion criteria and were consecutively enrolled in our hospital from January 2024 to September 2024, while 22 hips from 11 volunteers matched for age and sex were collected as controls. The study was approved by the Ethics Committee of the *** (KYLL-2022-1137) and performed according to the ethical standards established in the 1964 Declaration of Helsinki and its later amendments. All the participants provided written informed consent. Patients who satisfied the following criteria were included: were diagnosed with ONFH (stages I–IV, according to the Association Research Circulation Osseous, 2019 ^[15]^) by clinical appearance, plain radiography, or MRI; had no history of other hip disease or surgery; and could complete the questionnaire Short-Form-12-item Health Survey Version 2 (SF12) and the examination of ROM of the hip. Participants who met one of the following criteria were excluded: had experienced other hip disorders or surgery, and could not undergo MRI and evaluation of SF12 and ROM.

### 2.2. MRI assessment

**Figure 1.**
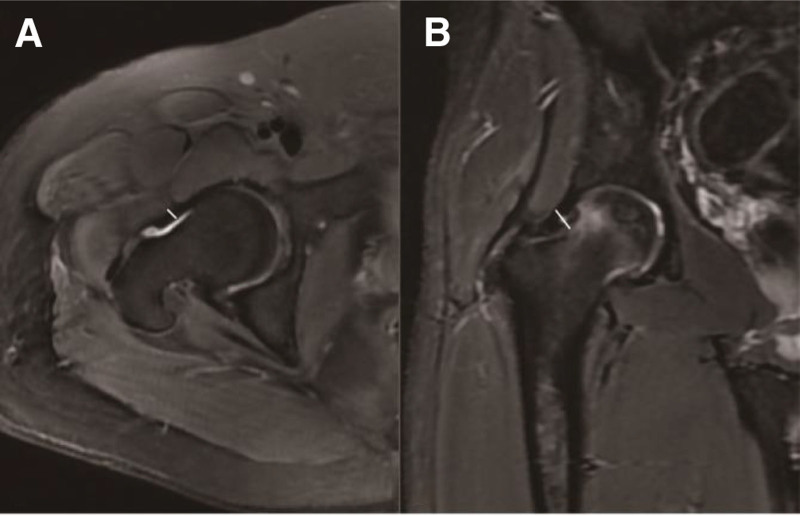
(A) Measurement of the thickness of the anterior capsule at 3:00 on an oblique axial MR image; (B) measurement of the thickness of the anterior capsule at 12:00 on an oblique coronal MR image. MR = magnetic resonance.

**Figure 2.**
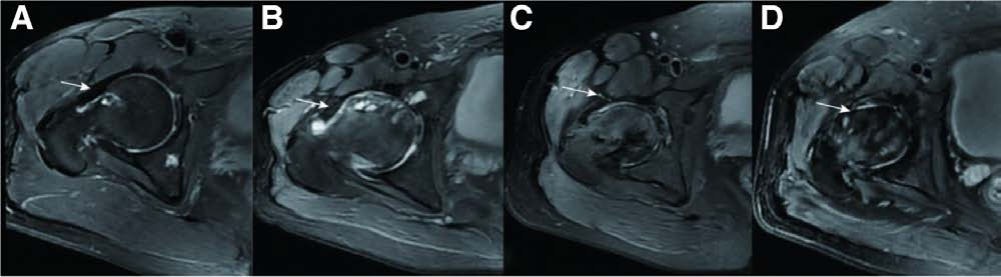
MRI classification of morphological alterations of the anterior capsule. (A) Grade 0, regular capsule without a hybrid signal in stage Ⅰ; (B) grade 1, local dotted high signal within the anterior capsule (edema) in stage Ⅳ; (C) grade 2, linear high signal within the anterior capsule but sustaining integrity (delamination) in stage Ⅱ; (D) grade 3, continuity of the anterior capsule disrupted and confused the boundary in stage Ⅲ. MRI = magnetic resonance imaging.

All subjects underwent preoperative MRI in the supine position using a 3 Tesla scanner (GE Healthcare, USA) with a cardiac or body coil. The routine protocol for oblique axial and oblique coronal planes included the following parameters: repetition time, 4010 ms; echo time, 60 ms; number of excitation, 2; echo train length, 7; field of view, 180 mm; matrix, 320 × 256; and slice thickness, 5 mm. No contrast agent was administered. Images were reviewed using a picture archiving and communication system by 2 experienced musculoskeletal radiologists (Tao and Gong) blinded to the diagnosis and group assignment. As depicted by Rakhra,^[6]^ the anterior joint capsular thickness was measured at 12:00 and 3:00 on the oblique coronal and oblique axial planes, respectively, according to the clock-face nomenclature (Fig. 1). The capsular thickness was computed by measuring the low-signal-intensity substance in the capsule between the cortical and muscular sides. A T2-weighted image at the level of the mid-femoral neck in the oblique axial and coronal planes was selected. Morphological alterations of the anterior capsule were investigated according to the MRI classification of meniscal injuries^[16]^ (grades 0–3, as shown in Figure 2), similar to Bai.^[7]^ The joint fluid in the articular cavity can also be assessed at different stages using T2-weighted images. The 1st observer performed 2 measurements at 2-week intervals to determine the intrarater reliability, and the 2nd observer measured the interrater reliability.

### 2.3. QOL measures

QOL assessments encompassing physical and mental function, as well as ROM, were conducted for all participants. Generally, comprehensive and disease-specific assessments should be evaluated simultaneously. The Oxford Hip Score (OHS),^[17]^ a disease-specific assessment, and the Short-Form-12 Health Survey version 2 (SF12)^[18]^ a comprehensive assessment, are employed for the QOL questionnaire. The OHS, consisting of 12 items, assesses pain and daily activities, with scores ranging from 0 to 48 points; a higher score indicates a better QOL. SF12 is a concise version of the SF-36 and includes a physical component summary (PCS) and a mental component summary (MCS). The national standard was set at 50 points for both PCS and MCS, with higher scores reflecting a better QOL. The ROM of the hip joint was examined through flexion, abduction, adduction, internal rotation, and external rotation in the supine position, as well as extension in the prone position.^[19]^ Ultimately, the correlations between PCS, MCS, and ROM and the changes in the anterior capsule in terms of components and dimensions were determined.

### 2.4. Statistics

All statistical analyses were performed using SPSS 24.0 (SPSS, Inc., Chicago). Categorical variables are presented as numbers or frequencies, and continuous variables are presented as mean ± standard deviation. The intrarater and interrater reliabilities were determined using the interclass correlation coefficient for continuous variables and the kappa statistic for categorical variables with a 95% confidence interval and were graded according to the Landis and Koch score.^[20]^ One-way ANOVA was used to evaluate significant differences in capsular thickness, PCS, MCS, and ROM among the different stages. Chi-square test or Fisher exact test was performed for categorical variables, including morphological alterations of the anterior capsule. Correlation between dimensional changes and PCS, MCS, and ROM was analyzed using the Pearson correlation coefficient, while componential changes, PCS, MCS, and ROM utilize the Spearman correlation coefficient. *P* < .05 was considered to indicate statistical significance.

## 3. Results

### 3.1. General outcomes

There were 46 patients (20 males and 26 females; overall mean age, 43 years) and 83 hips (40 from the left side and 43 from the right side) met the inclusion criteria for ONFH. All patients were divided into 4 stages according to the 2019 Association Research Circulation Osseous classification of ONFH. Twenty-two hips from 11 volunteers (5 males and 6 females; mean age 38 years) matched for age and sex were also enrolled as controls. There were no statistically significant differences in age, sex, body mass index, or etiology according to stage. The intrarater and interrater agreement of the thickness and morphological measurements exceeded 0.80, indicating good-to-excellent reliability.

### 3.2. Alterations in thickness in the coronal and axial planes

**Figure 3.**
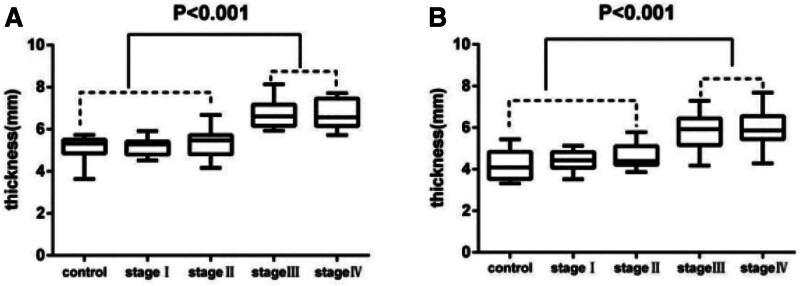
Boxplots illustrating that the thickness of the anterior capsule significantly differed (*P* < .001) between the early phases (control, stages Ⅰ, Ⅱ) and terminal phases (stage Ⅳ) in both the coronal plane (A) and axial plane (B); moreover, no significant differences were found within the early or terminal phases (*P* > .05).

Measurements of the anterior capsule on the 2 planes revealed significant differences among the various stages, especially between the early (I and II) and final (III and IV) stages (*P* < .001). In the coronal plane, early stage I and II patients had thinner capsules (5.2 ± 0.4 mm and 5.3 ± 0.7 mm, respectively) than did final stage III and IV patients (6.6 ± 0.6 mm and 6.7 ± 0.7 mm, respectively). On the axial plane, final stages Ⅲ and Ⅳ (5.8 ± 0.9 mm and 5.9 ± 0.9 mm) have thicker capsules compared to early stages Ⅰ and Ⅱ (4.4 ± 0.5 mm and 4.6 ± 0.6 mm). No significant differences were found in capsular thickness between early stage patients and controls in either plane. In the final stage, the mean thickness of the anterior capsule did not significantly differ between stage IV and III patients in either plane (Table 1, Fig. 3).

**Table 1:** Thickness (mm) of the anterior capsule in the coronal and axial planes and distribution (n) of morphological alterations in the anterior capsule.

	Stage Ⅰ	Stage Ⅱ	Stage Ⅲ	Stage Ⅳ	Control	*P*
Mean thickness						
Coronal plane	5.2 ± 0.4	5.3 ± 0.7	6.6 ± 0.6	6.7 ± 0.7	5.1 ± 0.5	<.001
Axial plane	4.4 ± 0.5	4.6 ± 0.6	5.8 ± 0.9	5.9 ± 0.9	4.2 ± 0.7	<.001
Classification						
Grade 0	14	9	3		22	<.001
Grade 1	3	6	6	3		
Grade 2		5	8	10		
Grade 3			6	9		

### 3.3. Distribution of morphological alterations in different stages

**Figure 4.**
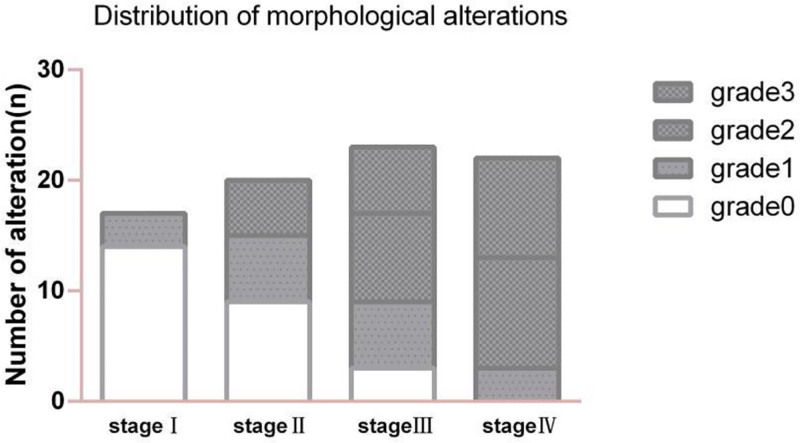
The illustration reveals the significant differences (*P* < .001) in morphologic characteristics between stages, with the parts filled with dark colourifying edema and delamination (grades 1–3) and the parts filled with blanks indicating normal color (grade 0). Moreover, an increasing trend in the incidence of edema and delamination can be observed in advanced stages.

The grading of morphological alterations in the anterior capsule revealed significant differences between the various stages (*P* < .001). Delamination and edema were more common in patients with stage III and IV disease (Table 1). The incidence of edema and delamination (grades 1–3) at each stage was 3, 11, 20, and 22, respectively. The percentages of the total number of disorders at each stage were 17.64%, 55%, 86.95%, and 100%, respectively (Fig. 4). The liquid alterations did not differ among the stages.

### 3.4. Measurement of QOL and its relationship with alterations of anterior capsule

OHS showed significant differences among the stages (*P* < .001), with an aggravative evolution of the stage accompanied by a worse score. Within different stages, the control and stage I groups acquired better scores than stages II, III, and IV (*P* < .001). No significant difference was observed in the early (control and I, *P* = .53) or terminal stages (III and IV, *P* = .35). Both PCS and MCS showed significant differences among the stages (*P* < .001), with the trend of earlier stages accompanying a better score. Among the different stages, the control and stage I groups acquired better scores than the stages II, III and IV groups (*P* < .001). No significant difference has been revealed within the early stages (PCS, control, and I, *P* = .74). MCS, control and I; *P* = .88), and terminal stages (PCS, III, and IV; *P* = .86). MCS, III and IV, *P* = .52) (Table 2). The correlation between anterior capsule thickness and OHS, PCS, and MCS had a moderately negative association in either plane, similar to morphological alterations. (*P* < .001). ROM had a moderately negative correlation with thickness in both planes and morphological alterations, except adduction (Table 3).

**Table 2:** Measurement of QOL in different stages.

	Stage Ⅰ	Stage Ⅱ	Stage Ⅲ	Stage Ⅳ	Control	*P*
OHS	40.0 ± 4.6	34.1 ± 4.9	26.4 ± 5.5	24.9 ± 5.9	41.1 ± 5.9	<.001
SF12						
PCS	41.2 ± 9.1	37.4 ± 8.2	26.3 ± 7.6	25.9 ± 7.1	42.8 ± 8.4	<.001
MCS	49.8 ± 7.2	42.9 ± 10.5	30.5 ± 7.6	28.8 ± 8.1	50.2 ± 8.0	<.001
ROM (°)						
Flexion	117.8 ± 8.4	112.2 ± 9.4	102.9 ± 10.2	84.8 ± 15.1	119.1 ± 11.6	<.001
Extension	16.7 ± 4.0	15.0 ± 4.5	11.0 ± 3.7	8.8 ± 4.2	16.9 ± 3.8	<.001
Abduction	29.4 ± 5.3	26.8 ± 7.10	21.5 ± 6.0	17.3 ± 5.3	30.3 ± 5.6	<.001
Adduction	15.3 ± 4.8	14.2 ± 5.4	10.9 ± 4.1	9.8 ± 4.9	15.9 ± 4.4	<.001
Internal rotation	41.5 ± 6.5	39.0 ± 6.5	27.7 ± 4.9	18.4 ± 5.0	42.2 ± 5.3	<.001
External rotation	41.1 ± 5.0	39.3 ± 6.1	27.3 ± 5.5	21.8 ± 5.2	41.7 ± 6.4	<.001

MCS = mental component summary, OHS = Oxford Hip Score, PCS = physical component summary, QOL = quality of life, ROM = range of motion, SF12 = Short-Form-12-item Health Survey Version 2.

**Table 3:** Correlation between QOL and alternations in anterior capsule.

	OHS	SF12		Range of motion					
		PCS	MCS	Fle	Ext	Abd	Add	I-R	E-R
Thickness									
Coronal	-0.57*	-0.51*	-0.55*	-0.51*	-0.43*	-0.46*	-0.19**	-0.54*	-0.53*
Axial	-0.54*	-0.56*	-0.60*	-0.50*	-0.45*	-0.56*	-0.01**	-0.64*	-0.61*
Morphology	-0.64*	-0.60*	-0.65*	-0.57*	-0.48*	-0.51*	-0.16**	-0.67*	-0.64*

MCS = mental component summary, OHS = Oxford Hip Score, PCS = physical component summary, QOL = quality of life, ROM = range of motion, SF12 = Short-Form-12-item Health Survey Version 2.

* *P* < .05.

** *P* > .05.

## 4. Discussion

This study characteristically determined preoperative alterations in the thickness and morphology of the hip anterior capsule in patients with ONFH. The outcome revealed that the thickness of the anterior capsule in the present study was consistent with prior radiological and anatomical investigations.^[6,21]^ The thicknesses in stages III and IV were significantly greater than those in stages I and II and the control in both the coronal and axial planes. A significantly thinner capsule was also observed on the axial plane than on the coronal plane at all stages, although no significant difference was found. Morphological alterations, including edema and delamination, were frequently visualized in the advanced stages, especially stages III and IV. The differences in the distributions of the various stages were significant. A moderately negative correlation between thickness and morphological alterations with OHS, SF12, and ROM also showed worse QOL in the terminal stages.

Patients with ONFH inevitably experience several characteristic side effects, such as hip pain, hypomobility, and even disability, and have several specific histopathological changes and eventual collapse of articular cartilage and osteoarthritis.^[22]^ Therefore, possible reasons for the outcomes observed in the present study are the pathological procedures used for ONFH. The histological changes observed in patients with ONFH in stages I and II included various types of osteocyte death and inflammatory reparative processes around the necrotic region. In contrast, the appearance of the femoral head did not change.^[3]^ Wang et al^[4]^ conducted experimental and microcomputed tomography evaluations of specimens from patients with ONFH in the early stages and revealed that the trabeculae were fractured around the necrotic area, a sclerotic band was generated with increased osteoblast activity, and the surface of the femoral head was significantly unaltered. Previous research has shown that the histopathological evolution of ONFH in the early stages, such as ischemia, osteocyte necrosis, and sclerotic area generation, is constrained within the femoral head without penetration of the articular surface of the femoral head. As a result, pathological development restricted within the head may not interfere with the joint capsule between the early stage and control stages.

Progressive stimulation after collapse of the femoral head and secondary osteoarthritis in stages III and IV can result in additional changes in adjacent tissues.^[3,23]^ Kato et al^[23]^ reported that joint biomarkers in synovial fluid, such as antigenic keratan sulfate, are significantly different in ONFH compared with osteoarthritis of the hip. Fondi et al^[24]^ reported the detection of severely deformed head shapes and osteochondral debris in the capsule and synovium in advanced stages. Therefore, deformed head and osteochondral debris stimulation could induce adjacent pathological capsular alterations and changes in biomechanics with increased stress load can lead to adaptive capsular thickening.^[9]^ Prior research has verified that the anterior-superior aspect of the femoral head is the most specific region of the body. This region is loaded with the most significant force during motion,^[25]^ after which the head initially fractures and collapses.^[26]^ Correspondingly, in this study, the patients were evaluated in the axial and coronal planes, which are the anterior and superior aspects of the femoral head, respectively. Other studies related to mechanical changes and diseases, such as femoroacetabular impingement, have also shown that the anterosuperior capsule is thicker than that in other regions.^[27]^ The authors suggested several factors, including stimulation of the deformed head, adaptive changes in biomechanics due to osteoarthritis, and predilection sites for the most significant load and collapse, all of which can contribute to the greater alteration of the anterior capsule observed in advanced stages. Moreover, severe morphological alterations of the anterior capsule in the terminal stage, such as edema and delamination, are consistent with progressive pathological changes in the bone structure. The author advocates the possible explanation of delamination in capsules, proposed by Hui,^[7]^ that the anterior capsule consists of numerous histological layers and ligaments with different orientations and mechanical characteristics and can experience adaptive changes according to the disease.

The hip joint capsule, which is composed of 3 fibrous ligaments (iliofemoral, ischiofemoral, and pubofemoral), covers the region between the acetabulum and proximal femur to constrain hypermobility and stabilize the hip joint.^[28]^ Specifically, the iliofemoral ligament restrains external rotation in flexion and internal rotation in neutral and extension, which the pubofemoral ligament restricts external rotation during extension.^[29]^ Moreover, both the iliofemoral ligament and PEL comprise the intact anterior capsule. Previous research has proposed the thickening of the anterior capsule further restrains hip motion.^[30]^ Therefore, the physiological foundation and prior study proposals may elucidate the reason for the moderate negative correlation between thickness and morphological alterations of the anterior capsule and hip ROM. Stimulation due to edema and delamination and limitation of ROM may severely affect daily activities, namely QOL, as revealed in this study through a negative correlation between the thickness and morphological alterations of the anterior capsule and OHS and SF12. However, the exact reason for the outcomes of this study remains for further investigations to verify. It is worth noting that as the crucial structure for stabilizing the hip joint, pathoanatomic changes in the anterior capsule accompanied by an evolution of ONFH should raise more concern so as to deepen the understanding of pathological changes, explore the possible clinical meaning of this change, and even help the decision-making preoperatively.

Based on the findings of this study, pathological changes in the anterior joint capsule during the advanced stage of ONFH (such as thickening, edema, and layering) are significantly associated with diminished QOL, underscoring the importance of early intervention. Although current evidence does not sufficiently support the efficacy of non-surgical treatments, including pharmaceutical or physical therapy, in preventing femoral head collapse or delaying the need for joint replacement, these approaches still hold value in pain alleviation.^[31]^ Among various hip-preserving surgical options, core decompression combined with bone graft biomaterial filling and/or structural support is recommended as a conventional treatment for early stage ONFH. This procedure effectively removes necrotic bone, reduces intraosseous pressure, and improves femoral head blood supply, thereby enhancing osteogenesis and therapeutic outcomes.^[32]^ With advances in research, emerging therapeutic strategies such as stem cell therapy and exosome-based treatments have been investigated and have demonstrated promising results in the management of ONFH.^[33,34]^

This study had several limitations. First, a larger sample size is needed to eliminate bias. Moreover, we measured capsular thickness using clock-face nomenclature in the MR scanner. The lack of a uniform assessment of the anterior capsule and numerous measurements are likely to generate various outcomes and conclusions. Additionally, morphological alterations in the anterior capsule were observed via meniscal injury according to MRI; therefore, there is an insufficient consensus. Finally, pathological assessments depend only on MRI imaging signals without histochemical analysis and intraoperative validation.

## 5. Conclusion

Thicker alterations, aggravated edema, and delamination of the hip anterior capsule can be observed on MRI in patients with stages III and IV ONFH. Thickness, edema, and delamination of the hip anterior capsule were negatively associated with ROM and QOL.

## Acknowledgments

Dr Li collaborated closely with the manuscript draft. Dr Fan provided invaluable support for the statistical data analysis. Dr Ma helped collect demographic and radiological data. Throughout the research and writing process, Ge ensured adherence to the highest standards and guidelines.

## Author contributions

**Data curation:** Zhiqiang Wang.

**Formal analysis:** Zhiqiang Wang.

**Funding acquisition:** Zhaohui Ge.

**Investigation:** Bobo Wang.

**Methodology:** Anli Shi, Zhaohui Ge.

**Writing – original draft:** Anli Shi, Bobo Wang.

**Writing – review & editing:** Zhaohui Ge.
